# *De Novo* Sequencing and Assembly Analysis of the *Pseudostellaria heterophylla* Transcriptome

**DOI:** 10.1371/journal.pone.0164235

**Published:** 2016-10-20

**Authors:** Jun Li, Wei Zhen, Dengkai Long, Ling Ding, Anhui Gong, Chenghong Xiao, Weike Jiang, Xiaoqing Liu, Tao Zhou, Luqi Huang

**Affiliations:** 1 Guiyang University of Chinese Medicine, Guiyang 550025, China; 2 National Engineering Research Center of Miao’s Medicines, Guiyang 550025, China; 3 State Key Laboratory Breeding Base of Dao-di Herbs, National Resource Center for Chinese Materia Medical, China Academy of Chinese Medical Sciences, Beijing 100700, China; Shanghai Ocean University, CHINA

## Abstract

*Pseudostellaria heterophylla* (Miq.) Pax is a mild tonic herb widely cultivated in the Southern part of China. The tuberous roots of *P*. *heterophylla* accumulate high levels of secondary metabolism products of medicinal value such as saponins, flavonoids, and isoquinoline alkaloids. Despite numerous studies on the pharmacological importance and purification of these compounds in *P*. *heterophylla*, their biosynthesis is not well understood. In the present study, we used Illumina HiSeq 4000 sequencing platform to sequence the RNA from flowers, leaves, stem, root cortex and xylem tissues of *P*. *heterophylla*. We obtained 616,413,316 clean reads that we assembled into 127, 334 unique sequences with an N50 length of 951 bp. Among these unigenes, 53,184 unigenes (41.76%) were annotated in a public database and 39, 795 unigenes were assigned to 356 KEGG pathways; 23,714 unigenes (8.82%) had high homology with the genes from *Beta vulgaris*. We discovered 32, 095 DEGs in different tissues and performed GO and KEGG enrichment analysis. The most enriched KEGG pathway of secondary metabolism showed up-regulated expression in tuberous roots as compared with the ground parts of *P*. *heterophylla*. Moreover, we identified 72 candidate genes involved in triterpenoids saponins biosynthesis in *P*. *heterophylla*. The expression profiles of 11 candidate unigenes were analyzed by quantitative real-time PCR (RT-qPCR). Our study established a global transcriptome database of *P*. *heterophylla* for gene identification and regulation. We also identified the candidate unigenes involved in triterpenoids saponins biosynthesis. Our results provide an invaluable resource for the secondary metabolites and physiological processes in different tissues of *P*. *heterophylla*.

## Introduction

*Pseudostellaria heterophylla* (Miq.) Pax, known as Hai Er Shen (HES) and false starwort belongs to the *Caryophyllaceae* family. The *Chung Yao Chi New Chinese Materia Medica* records the collection of HES plants since 1959 because of its local and ethnic use. *P*. *heterophylla* are distributed widely in the southern parts of China including Fujing, Jiangsu, Anhui, Shandong, Shanxi, Zhejiang, Jiangxi, Hubei, Shanxi, and Guangzhou provinces. The *P*. *heterophylla* is a mild tonic herb, weaker than *Panax ginseng* and popularly used in Traditional Chinese Medicines (TCM) products such as *Jiangzhong Jianweixiaoshi Tablets*, *Composite Pseudostellaria granule*. The mitogenic fraction (PH-I) from the hot water extract of *P*. *heterophylla* has significant potent anti-tumor activities against Ehrlich ascites tumor (EAT) cells in mice *in vivo* but not *in vitro* by releasing the tumor necrosis factor (TNF) [1]. The ethyl acetate fraction extracted from the roots of *P*. *heterophylla* markedly reduced the number of coughs and prolonged the latent cough period in rat model of stable phase chronic obstructive pulmonary disease induced by cigarette smoke exposure [2].

Saponins in *P*. *heterophylla* (PHS) are primary bioactive compounds and consist of Pseudostellarinoside A and A-cutifolisde D, both of which are oleanyl-type saponins [3]. PHS extracts have significant anti-fatigue, anti-anoxia activities [4] and prevent cell membrane of H9c2 cell from oxidative injury via preventing increased oxidative stress [5]. The precursor for the biosynthesis triterpenoid saponins is 2,3-oxidosqualene, which is synthesized via the MVA pathway [6]. Oxidosqualene cyclase (OSC) catalyzes the cyclization of 2.3-oxidosqualene to produce various triterpene skeletons. Some of candidate genes involved in triterpene saponin biosynthesis were isolated from *P*. *quinquefolium* [7], *P*. *ginseng* [8], and *P*. *notoginseng* [9], but none were identified from *P*. *heterophylla*.

Tuberous roots or stem, are the primary medicinal plant organs of TCM plants such as P. *heterophylla* [10], *Fallopia multiflora* [11], *Panax notoginseng* [12], *Salvia miltiorrhiza* [13]. Chemical technology has helped identify the secondary metabolites including flavonoids, an isoquinoline alkaloid, terpenoids, and phenylpropanoid in these plants [3]; however, there have been no molecular studies on secondary metabolism pathways involved in their biosynthesis and degradation. Hua et al.[14] (2016) performed *de novo* sequencing and transcriptome analysis of *P*. *heterophylla* tuberous roots, but no transcriptomic and genomic information from the aboveground parts (leaf, stem, and flower) is available in the nucleotide databases of National Centre for Biotechnology Information (NCBI). Study of the molecular basis of traits related to saponin biosynthesis and secondary metabolism in *P*. *heterophylla* will facilitate its breeding and improvement. RNA-seq is a useful tool for studying the expressed transcripts in different tissues and stages [15]. In this study, we used Illumina HiSeq 4000 sequencing platform to sequence the mRNA of *P*. *heterophylla* from various tissues (flowers, leaves, stem, root cortex and xylem). A global transcriptome database of *P*. *Heterophylla* was constructed to identify the differentially expressed genes (DEGs) in different tissues and putative genes encoding the enzymes involved in the biosynthesis of triterpene saponins.

## Methods

### Plant materials and RNA extraction

*P*. *heterophylla* cultivar ‘Shitai 1’ was selected and grown in a commercial planting base in Sibing County, Guizhou Province, China. Five tissues (S1 Fig) were collected separately from three randomly selected individuals. After cleaning, all samples were cut into small pieces for RNA isolation, and partial materials were used for gene cloning and RT-qPCR. Total RNA was extracted following the instructions of the Transzol Plant RNA Extraction Kit (TransGen Biotech, Beijing, China). DNA contamination was removed using DNase I (Takara, Tokyo, Japan).

### cDNA library preparation and transcriptome sequencing

The construction of the cDNA libraries and the RNASeq was performed by Shanghai Majorbio Bio-pharm Technology Co., Ltd. (Shanghai, China). Firstly, mRNA were purified from 12 μg of total RNA from five tissues (flowers, leaves, stem, root cortex and xylem) by using Oligo(dT) magnetic beads, respectively. Then, the mRNA was disrupted into small fragments (200 ± 25 bp), which were used for the second-strand cDNA synthesis. These cDNA fragments were ligated with the Illumina paired-end sequencing adaptors. Finally, these libraries were sequenced on a paired-end flow cell using Illumina Hiseq 4000 platform. We obtained 5–8 GB of reads from each sample for *de novo* assembly.

### *De novo* assembly and Gene annotation

Before assembly, the adaptors and unknown nucleotides in raw reads were filtered with SeqPrep (https://github.com/jstjohn/SeqPrep) and Sickle software (https://github.com/najoshi/sickle). Then the high-quality clean reads from 15 samples were used for *de novo* assembly by Trinity software [16] (http://trinityrnaseq.sourceforge.net/). Finally, the redundant Trinity generated contigs were clustered to remove using TIGR Gene Indices Clustering Tools (TGICL) (http://www.tigr.org/tdb/tgi/software/).

ORF prediction was performed using the Markov model as described on http://trinityrnaseq.sourceforge.net/analysis/extract_proteins_from_trinity_transcripts.html). Then, the results were determined by Pfam database (http://pfam.xfam.org/). All unigenes were annotated using BLASTx by sequence comparison with various protein databases [i.e., Nr, Swissprot, Cluster of Orthologous Groups of proteins (COG), Kyoto Encyclopedia of Genes and Genomes (KEGG)], with an *e*-value cutoff of 1*e*-5. Function analysis of all unigenes was performed by subjecting to Gene Ontology (GO). Blast2GO program (https://www.blast2go.com/) was used to identify the GO term from all assembled unigenes. Finally, we used the WEGO software (http://wego.genomics.org.cn/) to perform GO function classification and determine the distribution of gene functions in *P*. *heterophylla* at the macromolecular level.

### Digital gene expression profiling

Gene expression profiles were performed using RSEM (http://deweylab.biostat.wisc.edu/rsem/). The reads per kb per million reads (RPKM) were used to normalize the expression levels for each gene in each tissue of *P*. *heterophylla*. The RPKM from all isoforms of the same gene were summed as the RPKM of that gene. Cluster 3.0 software (http://bonsai.hgc.jp/~mdehoon/software/cluster/) was used to normalize the expression level of triterpene saponins. Samples names are shown on the heat maps.

### Identification of the unigenes involved in triterpene saponins

The amino acid sequences of triterpene saponins were downloaded from NCBI and used for searching the P. *heterophylla* transcriptomic database. Putative genes of saponin biosynthesis in P. *heterophylla* were identified using the BlastP program with an *e*-value of 1e-10. The default hits were removed manually.

### Real-Time PCR verification

Total RNA was extracted from different tissues of *P*. *heterophylla* and first-strand cDNA synthesis was performed by the Reverse Transcriptional M-MLV (Takara, Japan). We used ABI 7500 real-time PCR system (Life Technologies, Carlsbad, CA, USA) to determine the expression by real time PCR. All reactions were performed using SYBR® Premix Ex Taq™ II (Takara Biotechnology, China) according to the procedure with ten-fold diluted cDNA as templates. Reactions were first incubated at 95°C for 30 s, followed by 40 cycles of amplification at 95°C for 5 s and then 60°C for 34 s, after a final cycle of amplification at 95°C for 15 s, 60°C for 1 min and 95°C for 15 s. The raw data were analyzed using ABI 7500 software, and expression levels were normalized to *PhACT2* gene (gi: KT363848) to minimize the variation of cDNA template contents. The expression level was shown using 2^−ΔCt^ method. The experiments were performed in three individual biological replications.

## Results

### Illumina paired-end sequencing data and *De novo* assembly

We obtained 87 Gb of sequencing data including 645,961,688 raw reads and 616,413,316 clean reads with the base average error rate below 0.02%. A brief overview of the transcriptome assembly statistics are shown in Table 1. We used the Trinity program for the *de novo* assembling of the clean data because *P*. *heterophylla* reference genome was not available. After removal of ambiguous reads and low-quality reads (Q20 < 20), 127,334 unique sequences were obtained from the cDNA library constructed from *P*. *heterophylla* flowers, stem, leaves, and tuberous roots of (Table 1). The Q20 percentage (sequencing error rate < 1%) and Q30 percentage were 98% and 93.81%, respectively. The GC percentage in ground parts (leaves, stem, and flowers) and underground parts (cortex and xylem of tuberous roots) were an average of 51.5% and 43.5%. The length of unigenes ranged from 201 to 82, 236 bp, with an N50 length of 951 bp. 48, 860 coding sequences were obtained from all *P*. *heterophylla* unigenes sequences, and 30, 396 CDSs (62.21%) were longer than 1000 bp.

**Table 1:** Sequence Summary of *P*. *heterophylla* tissues.

Organs	Samples	clean reads	Clean bases(Gb)	Error (%)	Q20 (%)	Q30 (%)	GC (%)
Flower	1_Z_H	32417586	4.6	0.0115	98.04	94	51.7
	3_Z_H	38552446	5.4	0.0115	98.02	93.99	50.37
	4_Z_H	30520166	4.3	0.0114	98.1	94.19	50.57
Stem	1_Z_J	42890328	6.1	0.0119	97.92	93.67	53.77
	3_Z_J	29240052	4.1	0.0123	97.72	93.2	50.96
	4_Z_J	40303560	5.7	0.0115	98.05	94.05	51.46
Leaf	1_Z_YD	33756994	4.8	0.0115	98.08	94.09	53.03
	3_Z_YD	37921060	5.4	0.0113	98.13	94.24	50.89
	4_Z_YD	35401430	5	0.0123	97.73	93.18	50.75
Root xylem	1_G_M	70142364	9.9	0.0119	98.01	93.73	43
	3_G_M	46857578	6.7	0.0116	98.13	93.99	43.31
	4_G_M	45086442	6.4	0.0115	98.16	94.06	43.11
Root cortex	1_G_P	39335332	5.6	0.012	97.99	93.62	43.69
	3_G_P	44758244	6.3	0.0125	97.79	93.11	43.97
	4_G_P	49229734	7	0.0116	98.18	94.08	43.93

(1) Reads sequencing from the left; (2) Reads sequencing from the right.

Q20: percentage of bases with a Phred value >20; Q30: percentage of bases with a Phred value >30.

### Functional annotation

**Fig 1:**
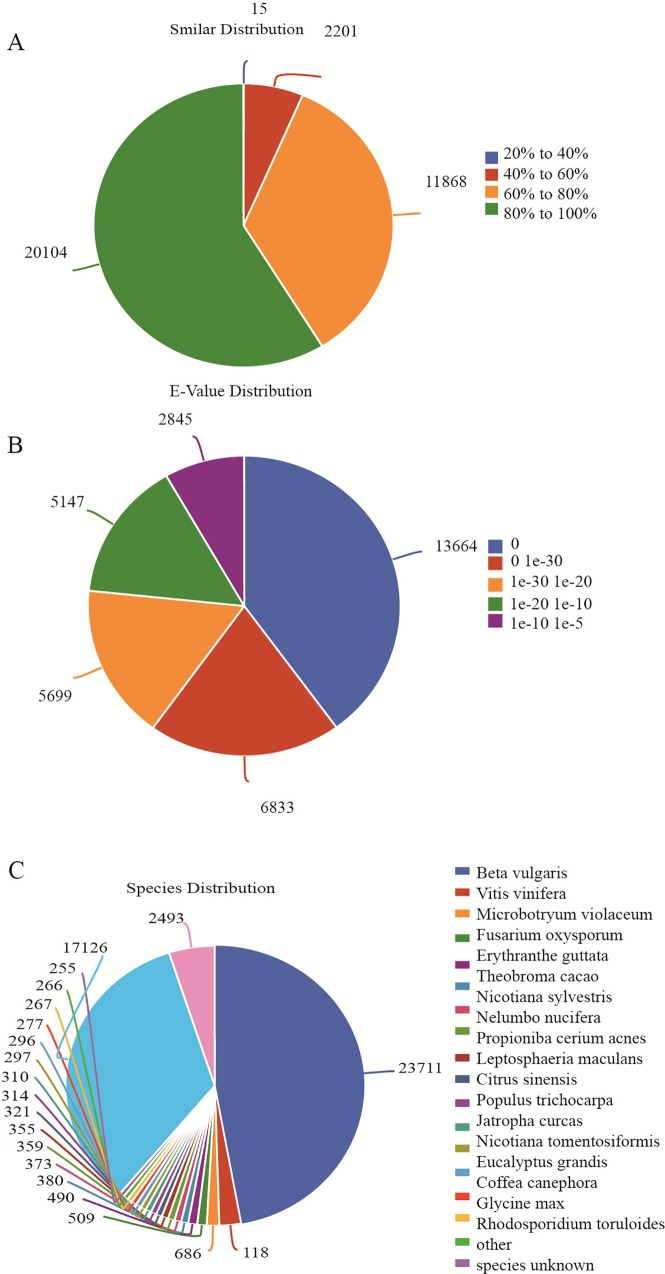
Species distribution of unigenes from *P*. *heterophylla*. a: Similarity distribution of top BLAST hits for each unigene; b: E-value distribution of BLAST hits with a cut off E-value of 1.0E−5; c: Species distribution for top BLAST hits in the Nr database.

Gene annotation showed that only a total of 52,937 unigenes (41.57%) had significant matches with the information from public databases. The annotation rate was much lower than those of previous reports [17, 18]. However, there are about 74,150 unigenes (58.43%) without any matches to known genes, and these unaligned genes may be specific genes and novel transcripts in P. *heterophylla*. Our results showed that 20,104 unigenes had high similarity (greater than 80%) in mapped sequences with Nr database and 20,497 unigenes (16.09%) had significant homology (e-value < 10^−30^) (Fig 1A and 1B). The mapping rates of unigenes against the Pfam, Swissprot, KEGG, String databases were 38.83%, 68.24%, 44.32% and 18.49%, respectively. The number of unigenes that were annotated in the unique database were as follows: 101 unigenes in the Pfam database, 90 unigenes in the SwissProt database, 36 unigenes in the KEGG database and 11,213 unigenes in the Nr database (Table 2). Species distribution analysis showed that only 23,711 unigenes (18.62%) had high homology with the genes from *Beta vulgaris*, followed by Vitis vinifera (1,182, 0.93%), *Theobroma cacao* (380, 0.30%), while 17,126 unigenes had high homology with sequences from other organisms (Fig 1C).

**Table 2:** Blast results of the assembled unigenes.

Database	Total unigenes	Annotated unigene	Percentage
Pfam	127334	20652	16.22%
Swissprot	127334	36291	28.50%
KEGG	127334	23572	18.51%
String	127334	16391	7.72%
Nr	127334	52937	41.57%

Unigenes showing high similarities with genes from *Microbotryum violaceum* (686 unigenes), *Fusarium oxysporum* (509 unigenes), *Leptosphaeria maculans* (321 unigenes), *Pseudomonas fluorescens* (272 unigenes), *Rhodosporidium toruloides* (255 unigenes) may belong to endophytes surviving in different parts of *P*. *heterophylla* [19]. Three unigenes from each species were validated by RT-PCR (S1 Table & S2 Fig).

### Functional classification

**Fig 2:**
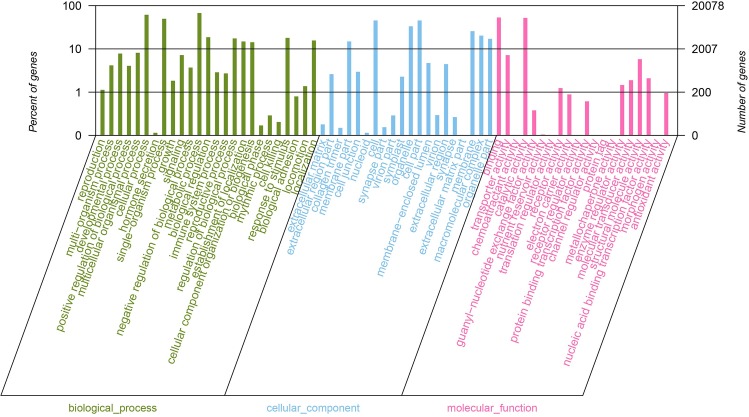
Gene Ontology classification of assembled unigenes. The unigenes were categorized into three main categories: biological process, cellular component, and molecular function.

We classified the functions of all unigenes using the Nr annotation and Gene Ontology (GO) classification (Fig 2, S2 Table). Moreover, we assigned 28, 210 unigenes to one or more gene ontology categories, 24,129 to molecular function, 15,544 unigenes to cellular component, and 23,751 unigenes to biological process. In the molecular function group, we found unigenes related to “catalytic activity” (15, 220, 53.95%) and “binding” (14,909, 52.85%). For the cellular component category, “cell” (7,660, 45.78%), “cell part” (7,659, 45.77%), “organelle” (5,601, 33.47%), “membrane” (4,380, 26.18%), “macromolecular complex” (3,485, 20.83%) represented the majority of unique sequences. Among molecular function category, unigenes assigned to “metabolic process” (11,388, 68.06%), “cellular process” (10, 343, 61.81%), and “single-organism process” (8,446, 50.47%) were the most abundant. A high percentage of genes were grouped into the “biological regulation” (3,217, 19.22%), “response to stimulus” (3,084, 18.43%), “regulation of biological process” (3,015, 18.02%), and “cellular component organization or biogenesis” (2, 461, 14.71%) categories.

**Fig 3:**
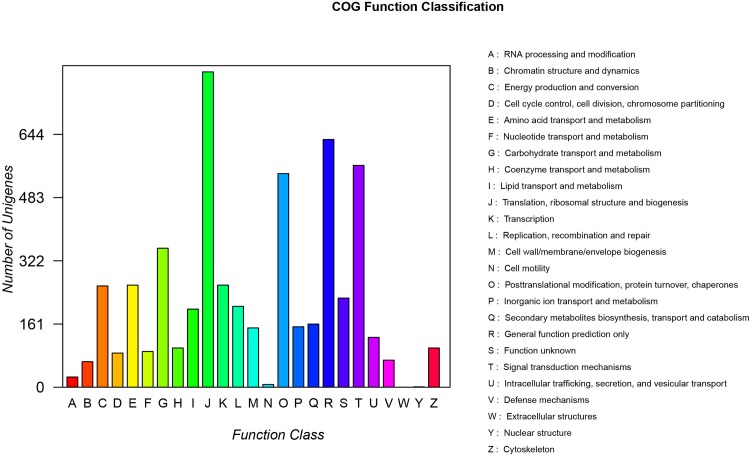
COG functional categories of *P*. *heterophylla*.

COG database was used for the function prediction and classification of all unigenes(Fig 3). In brief, 5,140 unigenes were grouped into 25 COG classifications. The largest group in the 25 COG categories was “translation, ribosomal structure and biogenesis” (803, 14.74%), followed by “general function prediction” (631, 11.58%), “signal transduction mechanisms” (565, 10.37%), and “posttranslational modification, protein turnover, chaperones” (544, 9.99%).

### KEGG classification

**Fig 4:**
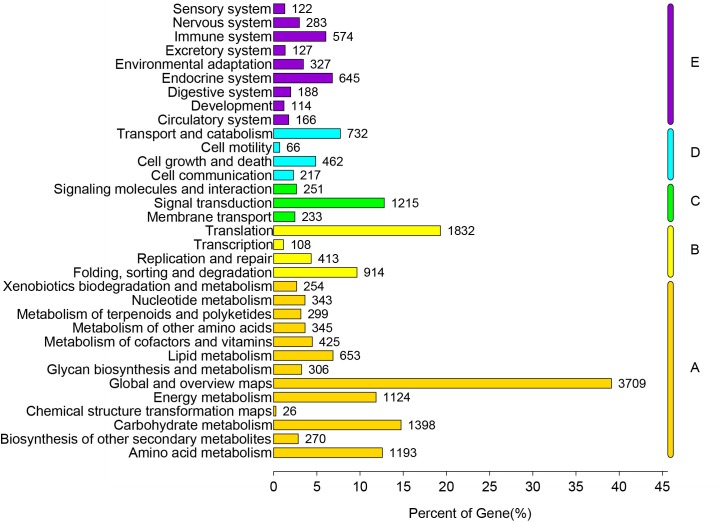
Pathway assignment based on the Kyoto Encyclopedia of Genes and Genomes (KEGG). (A) Classification based on metabolism categories, (B) Classification based on genetic information processing categories, (C) Classification based on environmental information processing categories, (D) Classification based on cellular processes categories, and (E) Classification based on organismal systems categories.

All unigenes were compared against KEGG for searching active biochemical pathways in *P*. *heterophylla* using BLASTx, with an e-value < 1e-10. We assigned 39, 795 unigenes to 356 KEGG pathways (Fig 4). “Ribosome” had the largest number of unigenes (1,075 unigenes) followed by “protein processing in endoplasmic reticulum” (404 unigenes), “oxidative phosphorylation” (390 unigenes), “glycolysis/gluconeogenesis” (315 unigenes), “endocytosis” (309 unigenes), “spliceosome” (287 unigenes). The metabolic pathways in our study were: “carbohydrate metabolism” (1,398 unigenes), “amino acid metabolism” (1,193 unigenes), “energy metabolism” (1,124 unigenes), “lipid metabolism” (653 unigenes), “metabolism of cofactors and vitamins” (425 unigenes), “metabolism of other amino acids” (345 unigenes), “nucleotide metabolism” (343 unigenes), “glycan of biosynthesis and metabolism” (306 unigenes), “metabolism of terpenoids and polyketides” (299 unigenes), and “biosynthesis of secondary metabolites” (270 unigenes). KEGG genetic information processing included “folding, sorting and degradation” (914 unigenes), followed by “replication” (413 unigenes) and “transcription” (108 unigenes). In the environmental information processing category, the most abundant subcategories were “signal transduction” (1,215 unigenes), “signaling molecules” and “interaction” (251 unigenes), and “membrane transport” (233 unigenes) (S3 Table).

### Differential Expression Analysis of *P*. *heterophylla*

**Fig 5:**
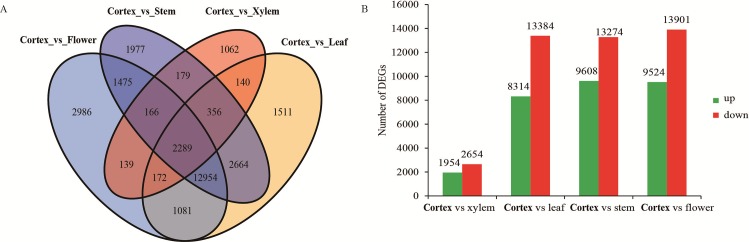
Venn diagrams of unigenes of three libraries and statistical analysis of the differentially expressed genes (DEGs). (A) Distribution of the unigenes of the three libraries; (B) The red columns indicate the up-regulated DEGs and the green columns represent the down-regulated DEGs in three pair-wise comparisons (FDR ≤ 0.001 and an absolute value of log 2 Ratio ≥ 1 was used as the significant threshold for DEGs).

We used our assembled data as a reference and compared the unigenes from different tissues of *P*. *heterophylla* (Fig 5A). A unigenes was regarded as a Differentially Expressed Gene (DEG) when FDR < 0.05 and log2|FC| > = 1. There were 32,095 DEGs between root cortex and xylem, of which 21,073 were down-regulated, and 11,022 were up-regulated (Fig 5B). There were 30,070 DEGs between root cortex and leaf, in which 18,495 were down-regulated and 11,575 up-regulated. Moreover, we identified 31,555 DEGs between root cortex and stem, 18,212 of which were down-regulated and 13,343 of which were up-regulated. Between root cortex and flower, 17,073 DEGs were down-regulated while 6,948 DEGs were up-regulated. Overall, we identified 2,289 common DEGs from the four comparison groups. Root cortex showed the highest number of upregulated unigenes among all tissues.

### GO enrichment analysis and KEGG enrichment analysis of DEGs in *P*. *heterophylla*

**Fig 6:**
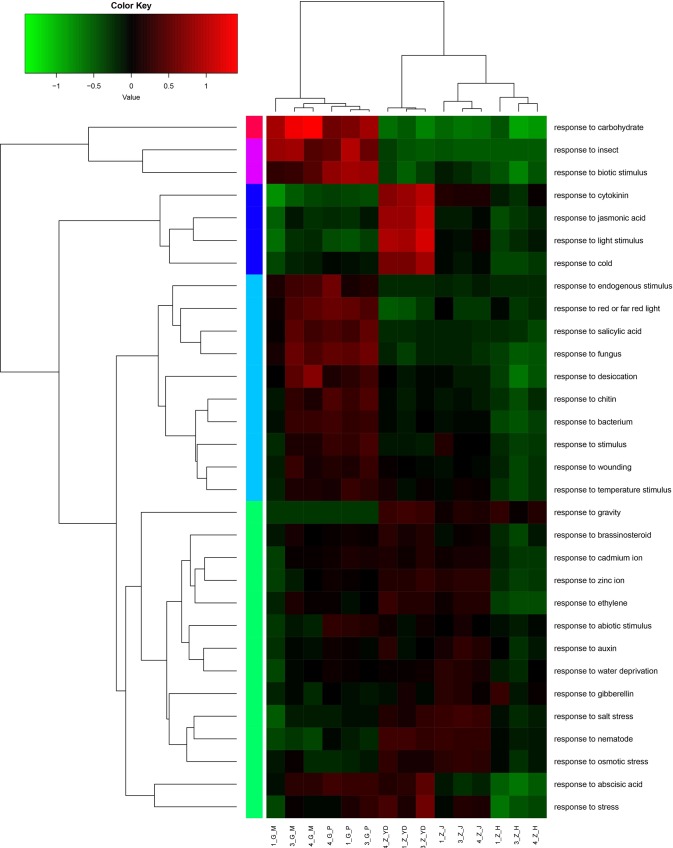
GO annotation of DEGs. The heat map shows 31 categories of DEGs in different tissues, leaf, stem, flower, root cortex and xylem included. Different colors indicated different expression levels. Green indicates down-regulated expression and red represents up-regulated expression. The heat map of all genes involved in each category was constructed using the log10 values of RPKM.1_G_M, 3_G_M, 4_G_M represents root xylem, 1_G_P, 3_G_P 4_G_P represents root cortex, 1_G_YD, 3_G_YD, 4_G_YD represents leaf, 1_G_J, 3_G_J, 4_G_J represents stem, 1_G_H, 3_G_H, 4_G_H represents flower from three individual plants.

The GO enrichment analysis and KEGG enrichment analysis elucidated the functional differences of DEGs from different *P*. *heterophylla* samples (S3 Fig). In GO enrichment analysis, the function was regarded as enriched if the corrected *p*-value of which was below 0.05. The result showed that the unigenes involved in “response to fungus”, “oligosaccharide metabolic process”, “defense response to other organism”, “chloroplast envelope”, “hydrolase activity, hydrolyzing O-glycosyl compounds”, “sucrose metabolic process” were enriched between root cortex and flower (S4 Fig). Highly enriched DEGs were involved in “response to auxin”, “root development”, “plastid thylakoid”, “chloroplast thylakoid”, and “chloroplast stroma” between root cortex and leaf (S5 Fig). The DEGs involved in “pollen development”, “gametophyte development”, “response to auxin”, “response to external stimulus”, and “thylakoid” were enriched between root cortex and stem (S6 Fig). Other highly enriched genes were related to “oxidation−reduction process”, “naringenin−chalcone synthase activity”, “flavonoid metabolic process”, “protein disulfide oxidoreductase activity” between root cortex and xylem (S7 Fig). Moreover, we also analyzed 31 response categories related to DEGs using the heatmap according to the total RPKM values of all the DEGs in each pathway (Fig 6). Among these categories, most categories were up-regulated in underground parts (root cortex and xylem), including “response to biotic stimulus”, “response to insect”, “response to carbohydrate”, “response to endogenous stimulus”, “response to fungus”, “response to bacterium” and “response to wounding”. The only categories active in leaf were “response to cytokinin”, “response to jasmonic acid”, “response to light stimulus”, “response to cold”. Our results showed that 11 out of 31 response pathways had up-regulated expression in both leaf and stem. These included “response to salt stress”, “response to brassinosteroid”, “response to auxin”, “response to water deprivation”, “response to gibberellin” and “response to salt stress”.

For a further study of DEGs, the KEGG database was used to search the significantly enriched biochemical pathway. Between root cortex and flower, the most significant enriched pathway was “plant hormone signal transduction”,which contained down-regulated DEGs in above-ground parts. Most of the DEGs that were involved in “plant-pathogen interaction”, “starch and sucrose metabolism”, “phenylpropanoid biosynthesis”, “alpha-Linolenic acid metabolism”, “circadian rhythm–plant”, “glycosylphosphatidylinositol (GPI)-anchor biosynthesis”, and “N-Glycan biosynthesis” were down-regulated. On the other hand, the DEGs involved in “diterpenoid biosynthesis”, “isoquinoline alkaloid biosynthesis”, “monoterpenoid biosynthesis”, “stibenoid diarylhepatanoid and gingerol biosynthesis”, “ubiquinone and other terpenoid-quinone biosynthesis”, “zeatin biosynthesis” were up-regulated (S8 Fig). We observed similar results in each underground parts (root cortex and xylem) compared to either aboveground parts (leaf, stem and xylem) in *P*. *heterophylla* (S9–S11 Figs).

**Fig 7:**
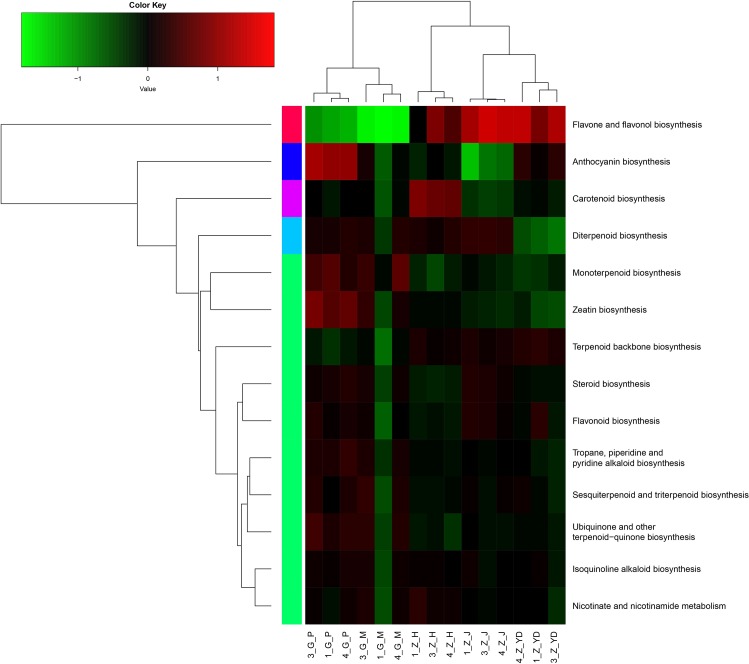
KEGG annotation of DEGs. The heat map shows 31 pathways of secondary metabolism in different tissues, including leaf, stem, flower, root cortex and xylem. Expression differences are shown in different colors. Red represents high expression and green represents the low expression. 1_G_M, 3_G_M, 4_G_M indicates root xylem, 1_G_P, 3_G_P 4_G_P indicates root cortex, 1_G_YD, 3_G_YD, 4_G_YD indicates leaf, 1_G_J, 3_G_J, 4_G_J indicates stem, 1_G_H, 3_G_H, 4_G_H represents flower from three individual plants.

We further used the heatmap to analyze 14 KEGG pathways involved in the biosynthesis of secondary metabolites in different tissues (Fig 7). Our analysis showed that 6 out of 14 pathways showed up-regulated expression in underground parts (root cortex and xylem) including “monoterpenoid biosynthesis”, “zeatin biosynthesis”, “tropane, piperidine and pyridine alkaloid biosynthesis”, “sesquiterpenoid and triterpenoid biosynthesis”, “ubiquinone and other terpenoid−quinone biosynthesis” and “isoquinoline alkaloid biosynthesis.” These results explain why tuberous root including root cortex and xylem is the principal medicinal part of *P*. *heterophylla*.

### Identification of genes involved in triterpenoids saponins biosynthesis of *P*. *heterophylla*

We identified 70 candidate genes in *P*. *heterophylla* including AACT (acetyl-CoA acetyltransferase), HMGS (HMG-CoA synthase), HMGR (HMG-CoA reductase), MVK (mevalonate kinase), PMK (phosphomevalonate kinase), MVD (mevalonate diphosphate decarboxylase), GGPS (geranylgeranyl pyrophosphate synthase), FPS (farnesyl diphosphate synthase), IDI (isopentenyl diphosphate isomerase), SS (squalene synthase), SE (squalene epoxidase), LuS (lupeol synthase), β-A28O (β-amyrin 28-oxidase) (S4 Table). 2,3-oxidosqualene is the the key enzyme at the first committed step and the skeleton of triterpenoids saponins in plants depends on its activity. Notably, three unigenes (c24484_g1, c60124_g1, c27529_g1) encoding lupeol synthase were identified from our transcriptome data, but none encoding β-amyrin synthase and dammarenediol-II synthase were identified.

**Fig 8:**
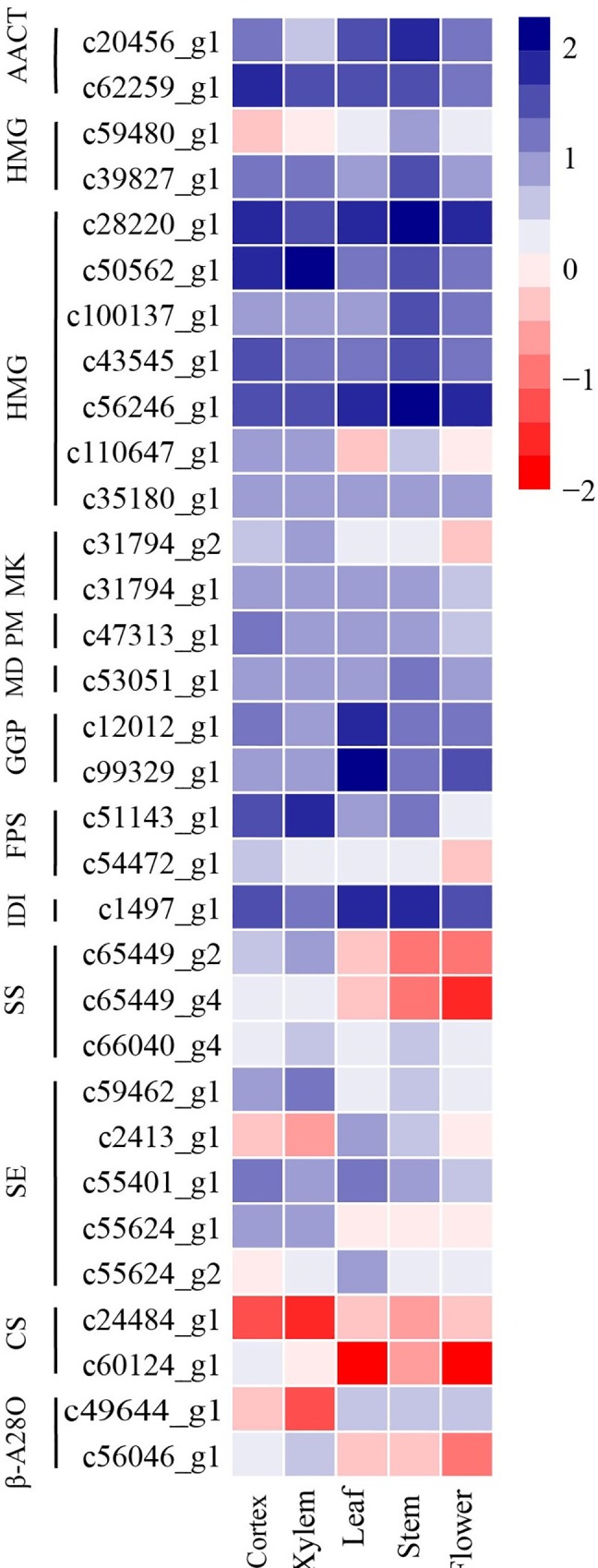
The expression profiles of unigenes involved in triterpene saponin biosynthesis of P. *Heterophylla*. Expression differences are shown in different colors. Red represents high expression and green represents the low expression.

**Fig 9:**
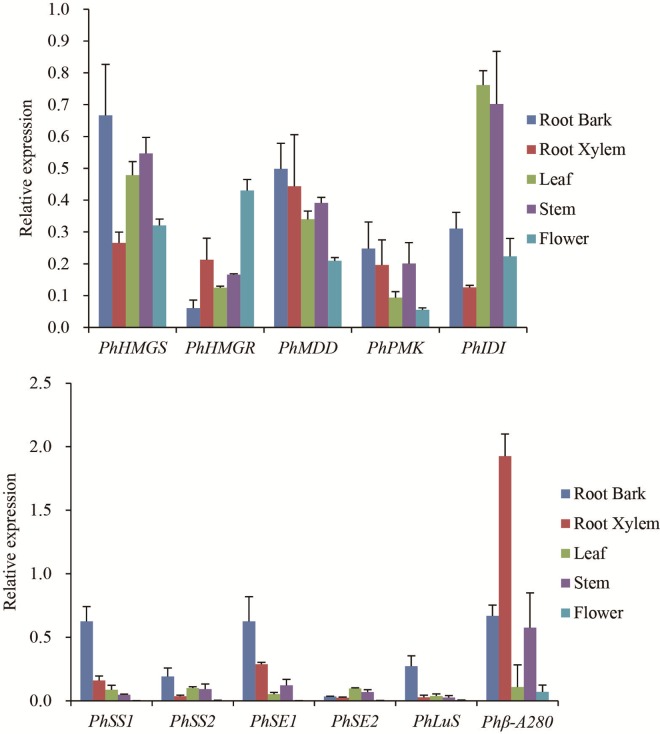
The expression validation of candidate genes in triterpene saponin biosynthesis of P. *heterophylla* by qRT-PCR. Error bars represent the mean (± SD) of three individual biologic experiments.

The heat map result showed that most unigenes encoding AACT, HMGS, MK, PMK, MDD and IDI, had high expression levels in flowers, leaves, stem, root cortex, and xylem (Fig 8). Some members of the gene family of HMGR, SE andβ-A28O were up-regulated in the root cortex and xylem while others were down-regulated. The unigenes encoding GGPS (c12012_g1, c99329_g1) and IDI (c1497_g1) were up-regulated specially in leaf and stem. Some investigated genes showed high expression levels in the root cortex and xylem such as FPS (c51143_g1, c54472_g1), SS (c65449_g2, c65449_g4, c66040_g4) and LuS (c60124_g1). The identification of genes involved in triterpenoids saponins biosynthesis may help explain the accumulation of saponins in different tissues of *P*. *heterophylla*. We validated the expression levels of 11 randomly selected genes using real-time PCR. The expression profiles of these unigenes were consistent with the transcriptomic data (Fig 9). Gene-specific primers were designed based on the gene sequences and are shown in S5 Table.

## Discussion

High throughput transcriptome sequencing has become a popular tool for sequencing non-model organisms such as *Gingko kernels* [20], *Rehmannia glutinosa* [21], *Gossypium hirsutum* [22], *Liriodendron chinense* [23], *Ramia* [24], and *Centella asiatica* [25]. We used Illumina HiSeq 4000 sequencing platform to sequence RNA from flowers, leaves, stem, root cortex, and xylem of *P*. *heterophylla*. The number of unigenes (127,334) identified in our study was much higher as compared with previous transcriptomic studies from Pseudostellariae *redix* [14]. Our data provides a useful resource for gene identification and regulation in different tissues of *P*. *heterophylla*.

Our transcriptomic data identified unigenes related to five endophytes. Three of these endophytes (*M*. *violaceum*, *L*. *maculans*, and *P*. *fluorescens*) are harmful for the development of plant organs [26–28]. *F*. *oxysporum* is an important replant disease pathogen in *Pseudostellaria heterophylla* rhizospheric soil [29] and also isolated from *Chamaecyparis lawsoniana* [30], *Quercus variabilis* [31] and *Ephedra fasciculate* [32]. Some active chemicals were previously purified from *F*. *oxysporum*, such as oxysporidinone (pyridine, anti-fungus) and beauvericin (cycle-peptide, anti-cancer) [33]. *R*. *toruloides* is an oleaginous yeast and used for lipid production [34]. The results of transcriptome data and reverse transcript PCR indicated that the transcripts of unigenes from *M*. *violaceum*, *P*. *fluorescens* and *R*. *toruloides* were detected in aboveground parts (leaf, stem and flower), the expression profiles of unigenes from *R*. *toruloides* and *L*. *maculans* were determined in underground parts (root cortex and xylem).These results suggest that endophytes may participate in the interaction between plants and microorganisms; and thus, provide a novel guideline for the planting of *P*. *heterophylla*.

The transcriptomic data from different tissues showed that most DEGs were either up-regulated in ground parts (leaf, stem, and flower) or underground parts (root cortex, and xylem) while a few DEGs showed special expression in certain tissues. The tuberous roots of sweet potato, cassava, and dahlia store nutrients, which permit survival from one year to the next. The formation of an enlarged area and secondary metabolic biosynthesis in the tuberous root is influenced by environment factors including fungus, bacteria, and wounding [35, 36]. In this study, these pathways were up-regulated both in root cortex and xylem. The pathways related to response to cytokinin, jasmonic acid, light stimulus, and cold were specially activated in the leaves. These results provided a better understanding of gene expression and regulation in different tissues of *P*. *heterophylla*.

The unigenes involved in triterpenoids saponins biosynthesis of *P*. *heterophylla* were identified. The cyclization of 2,3-oxidosqualene–catalyzed 2,3-oxidosqualene cyclases (OSCs)–is the first committed step in the triterpenoid saponins, which provides potential products [37]. Although the structure of saponins in *P*. *heterophylla* was similar to that of *P*. *vietnamensis* and *P*. *notoginseng*, we did not identify any unigenes encoding β-amyrin synthase and dammarenediol-II synthase. The OSCs in plants contain four genes coding β-amyrin synthase, dammarenediol-II synthase, lupeol synthase and cycloartenol synthase, respectively. Because of high similarities, these pentacyclic triterpene synthases may have evolved in a complicated order in triterpenoid saponin biosynthesis and sterol biosynthesis with a common progenitor [38]. The in vitro activities of OSCs were analyzed by expressing them in *Saccharomyces cerevisiae*, strains carrying OSC2 accumulated α-, β-, and δ-amyrin and strains carrying LuS accumulated α-amyrin and lupeol [39]. The above study suggested that 2,3-oxidosqualene in triterpenoids saponins biosynthesis of *P*. *heterophylla* may mainly rely on the activity of lupeol synthase. Moreover, the discovery of β-amyrin synthase requires a precise sequencing technology in the future.

Our qRT-PCR results and transcriptome data showed that two unigenes (c65449_g2, c65449_g4) encoding squalene synthase and two (c59462_g1, c55401_g1) encoding squalene epoxidase in triterpenoids saponins biosynthesis were up-regulated in both root cortex and xylem. Unigenes encoding GGPS (c99329_g1), IDI (c1497_g1), and MDD (c53051_g1) enzymes showed a high expression in both ground parts (leaf and stem) and underground parts (root cortex and xylem). Triterpene saponins can be extracted from underground parts (tuber root) and aerial parts (leaf and stem) [40] of P. *heterophylla*; however, these triterpene saponins may also accumulate in special tissues. Our study provides valuable information about pathways for the synthesis of triterpenoid saponins. Future studies involving isolation of key enzymes genes (OSCs) and their functional analysis are imperative for a complete understanding of the triperpenoid biosynthetic pathways.

## Supporting Information

S1 Fig Different tissues. (PDF)

S2 Fig Reverse transcript PCR determination of unigenes from endophytes. (PDF)

S3 Fig Heatmap_Cortex_Flower_Leaf_Stem_Xylem. (PDF)

S4 Fig Cortex_vs_Flower.DE.list.enrichment.detail.xls.go. (PDF)

S5 Fig Cortex_vs_Leaf.DE.list.enrichment.detail.xls.go. (PDF)

S6 Fig Cortex_vs_Stem.DE.list.enrichment.detail.xls.go. (PDF)

S7 Fig Cortex_vs_Xylem.DE.list.enrichment.detail.xls.go. (PDF)

S8 Fig Cortex_vs_Flower.pathway. (PDF)

S9 Fig Cortex_vs_Leaf.new.pathway. (PDF)

S10 Fig Cortex_vs_Stem.pathway. (PDF)

S11 Fig Cortex_vs_Xylem.pathway. (PDF)

S1 Table Primers of unigenes from endophytes used in reverse transcript PCR. (XLSX)

S2 Table unigene_GO.list.level234.stat. (XLSX)

S3 Table pathway_table. (XLSX)

S4 Table Candidate genes involved in triterpenoids saponins biosynthesis of *P*. *heterophylla*. (XLSX)

S5 Table List of gene-specific primers used in real time PCR. (XLSX)
