# Correction: *De Novo* Sequencing and Assembly Analysis of the *Pseudostellaria heterophylla* Transcriptome

**DOI:** 10.1371/journal.pone.0170134

**Published:** 2017-01-06

**Authors:** Jun Li, Wei Zheng, Dengkai Long, Ling Ding, Anhui Gong, Chenghong Xiao, Weike Jiang, Xiaoqing Liu, Tao Zhou, Luqi Huang

The second author’s name is incorrect. The correct name is: Wei Zheng. The correct citation is: Li J, Zheng W, Long D, Ding L, Gong A, Xiao C, et al. (2016) *De Novo* Sequencing and Assembly Analysis of the *Pseudostellaria heterophylla* Transcriptome. PLoS ONE 11(10): e0164235.

There is an error in the first sentence of the Methods section. The correct sentence is: *P*. *heterophylla* cultivar ‘Shitai 1’ was selected and grown in a commercial planting base in Shibing County, Guizhou Province, China.
