# Supplementary figures and images for: De Novo Sequencing and Assembly Analysis of the Pseudostellaria heterophylla Transcriptome
Source: PLoS One. 2016 Oct 20;11(10):e0164235. doi: 10.1371/journal.pone.0164235 (PMC5072632; doi:10.1371/journal.pone.0164235)

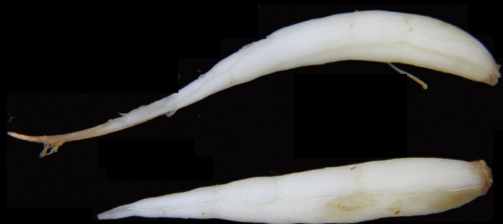

Root xylem

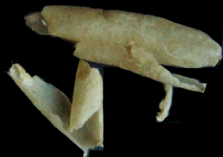

Root cortex

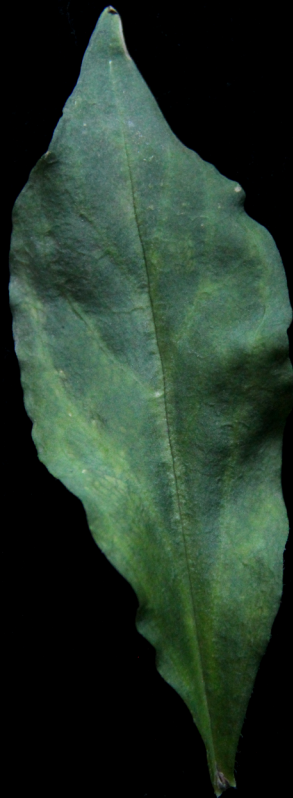

Leaf

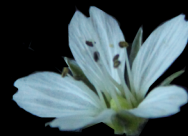

Flower

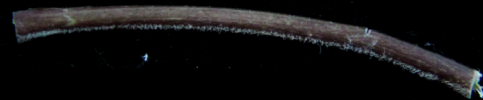

Stem

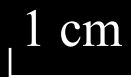

Supplement: S1 Fig — (PDF) [file pone.0164235.s001.pdf]

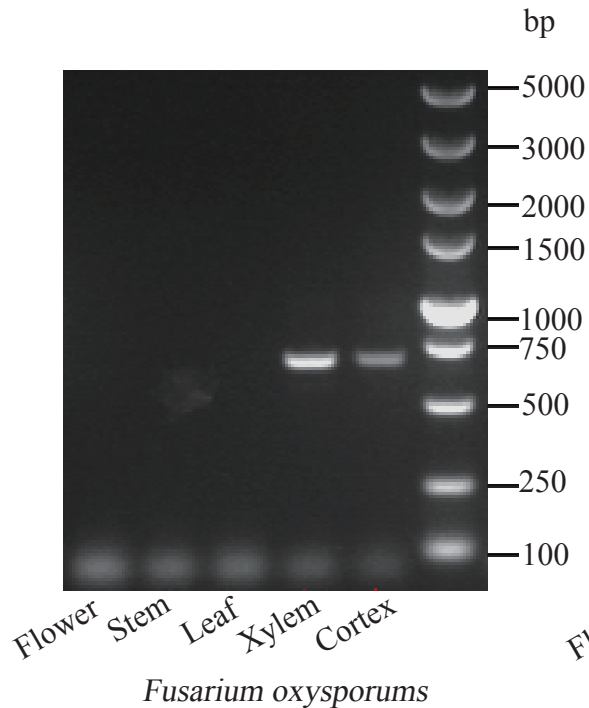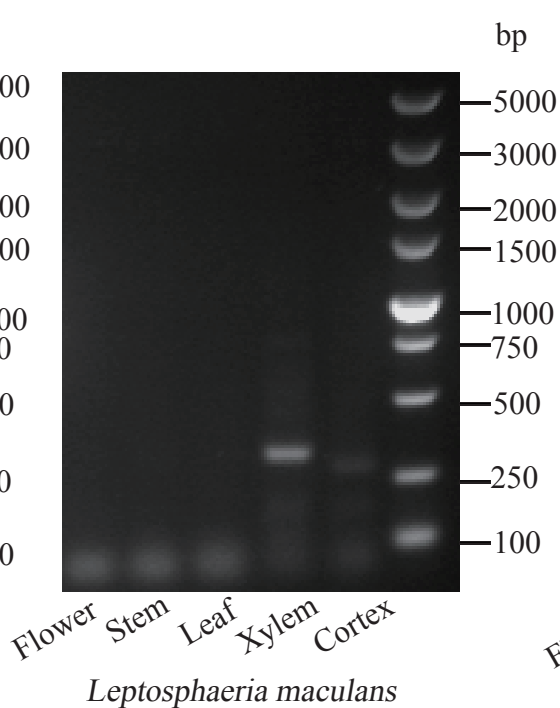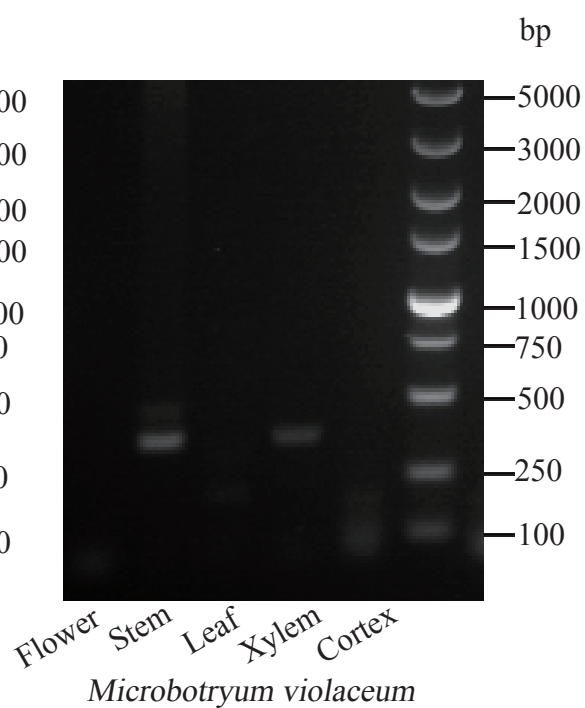

Supplement: S2 Fig — (PDF) [file pone.0164235.s002.pdf]

Color Key

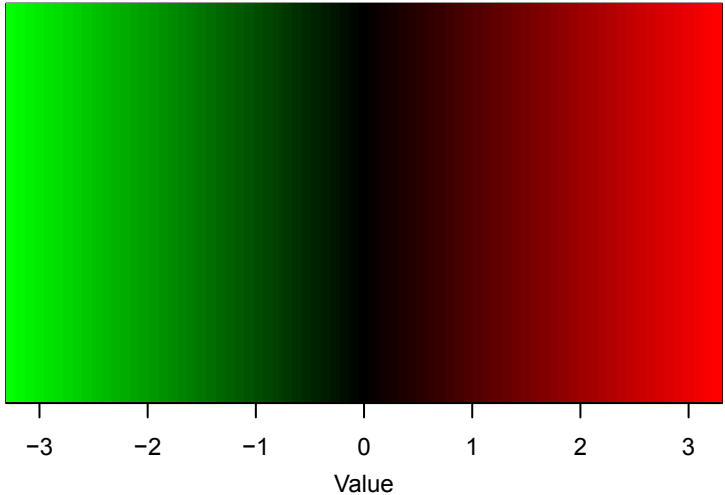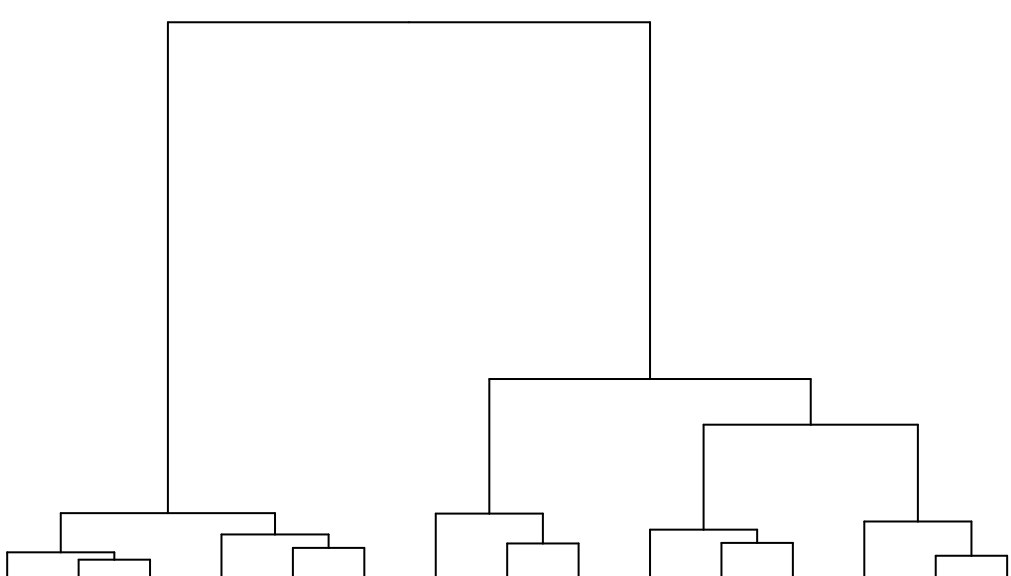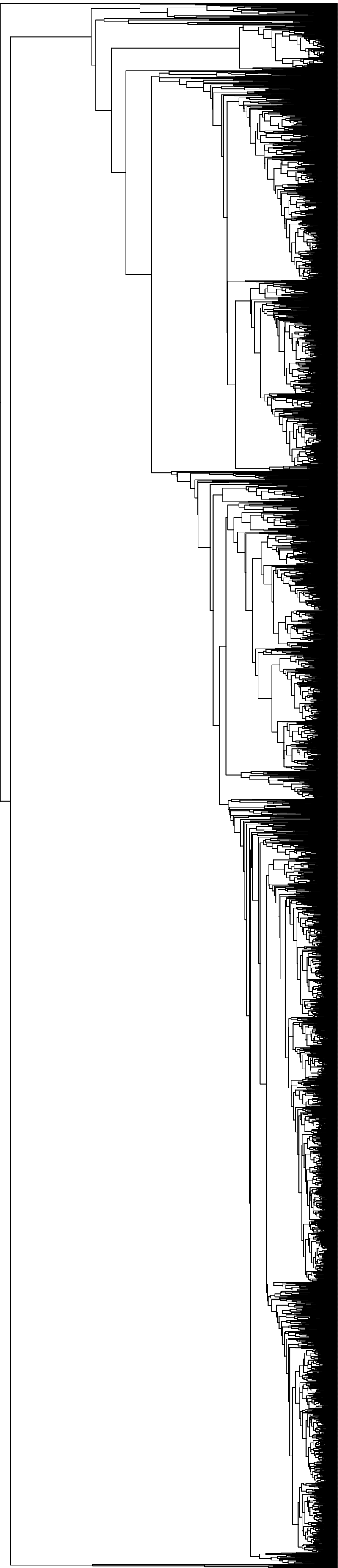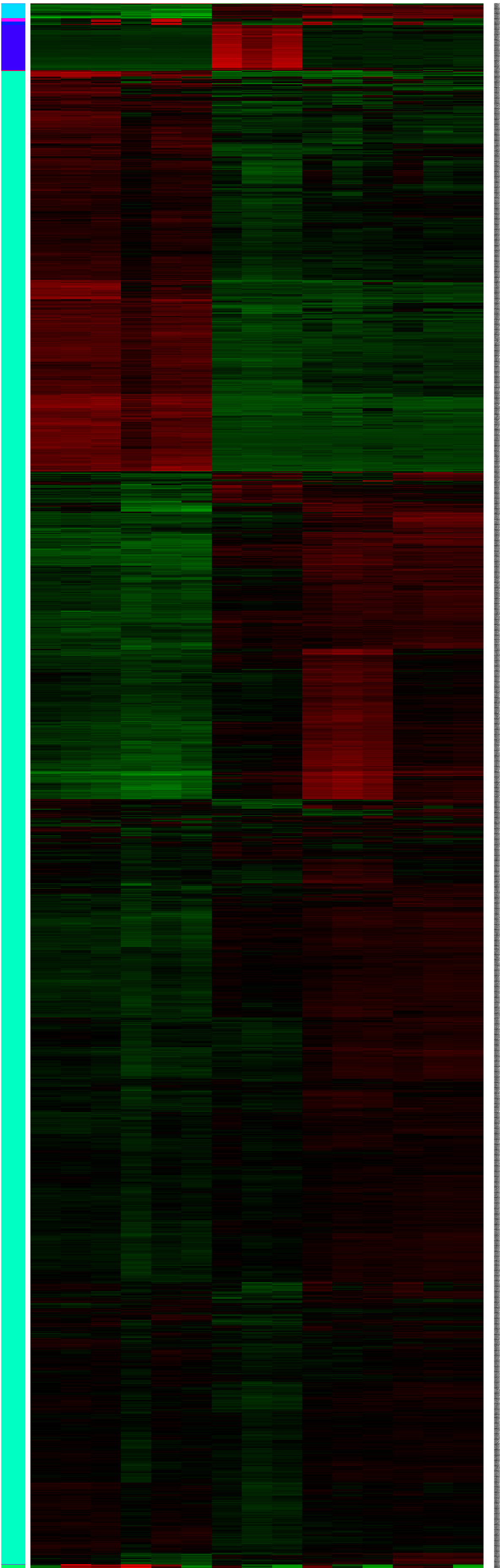

4\_G\_P  
1\_G\_P  
3\_G\_P  
1\_G\_M  
3\_G\_M  
4\_G\_M  
1\_Z\_H  
3\_Z\_H  
4\_Z\_H  
1\_Z\_YD  
3\_Z\_YD  
4\_Z\_YD  
1\_Z\_J  
3\_Z\_J  
4\_Z\_J

Supplement: S3 Fig — (PDF) [file pone.0164235.s003.pdf]

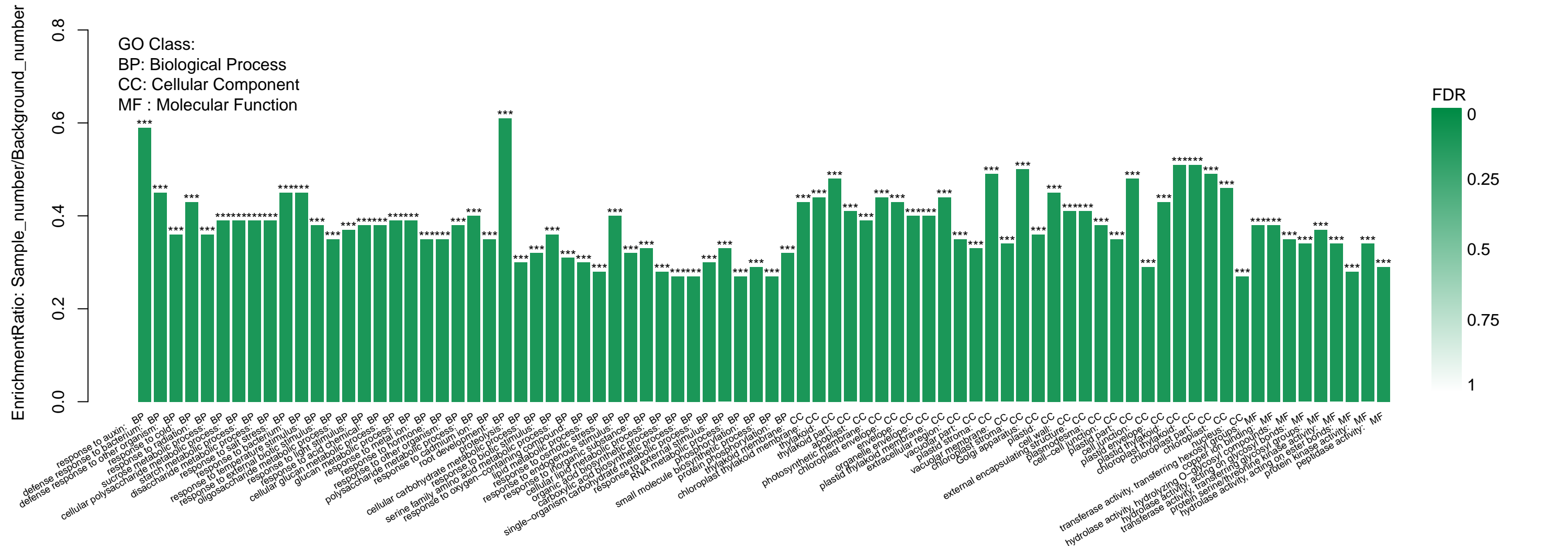

Supplement: S5 Fig — (PDF) [file pone.0164235.s005.pdf]

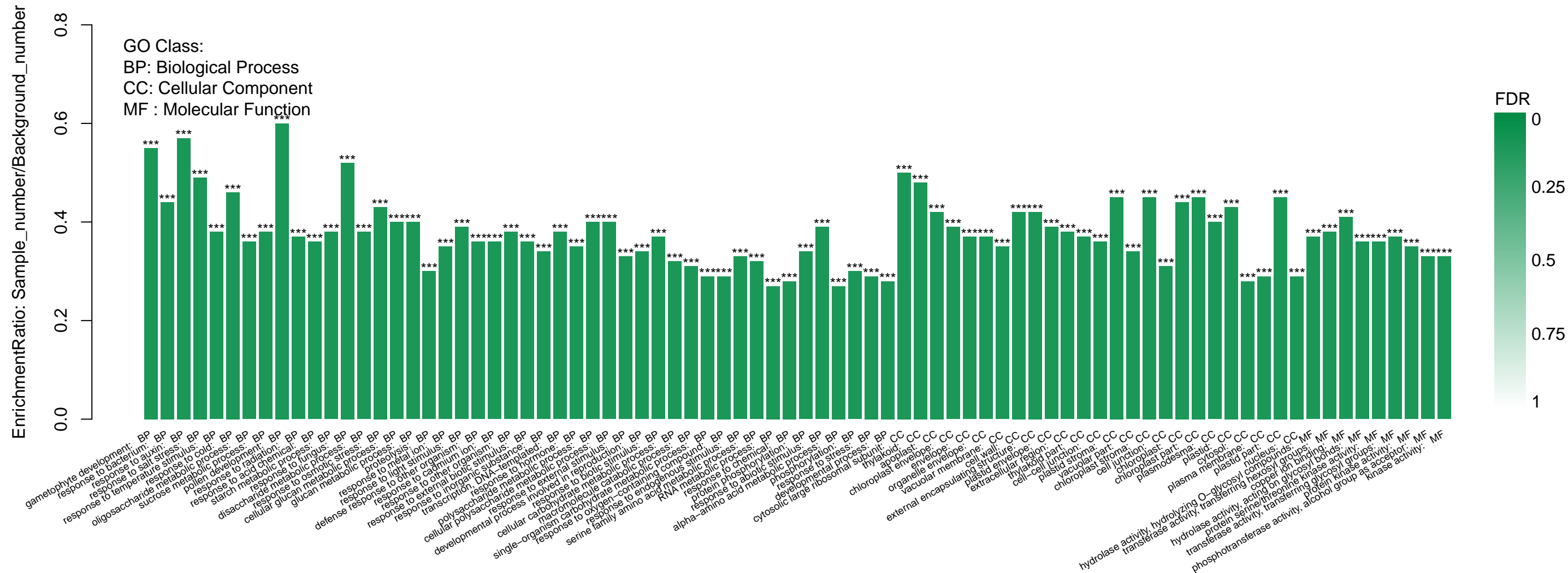

Supplement: S6 Fig — (PDF) [file pone.0164235.s006.pdf]

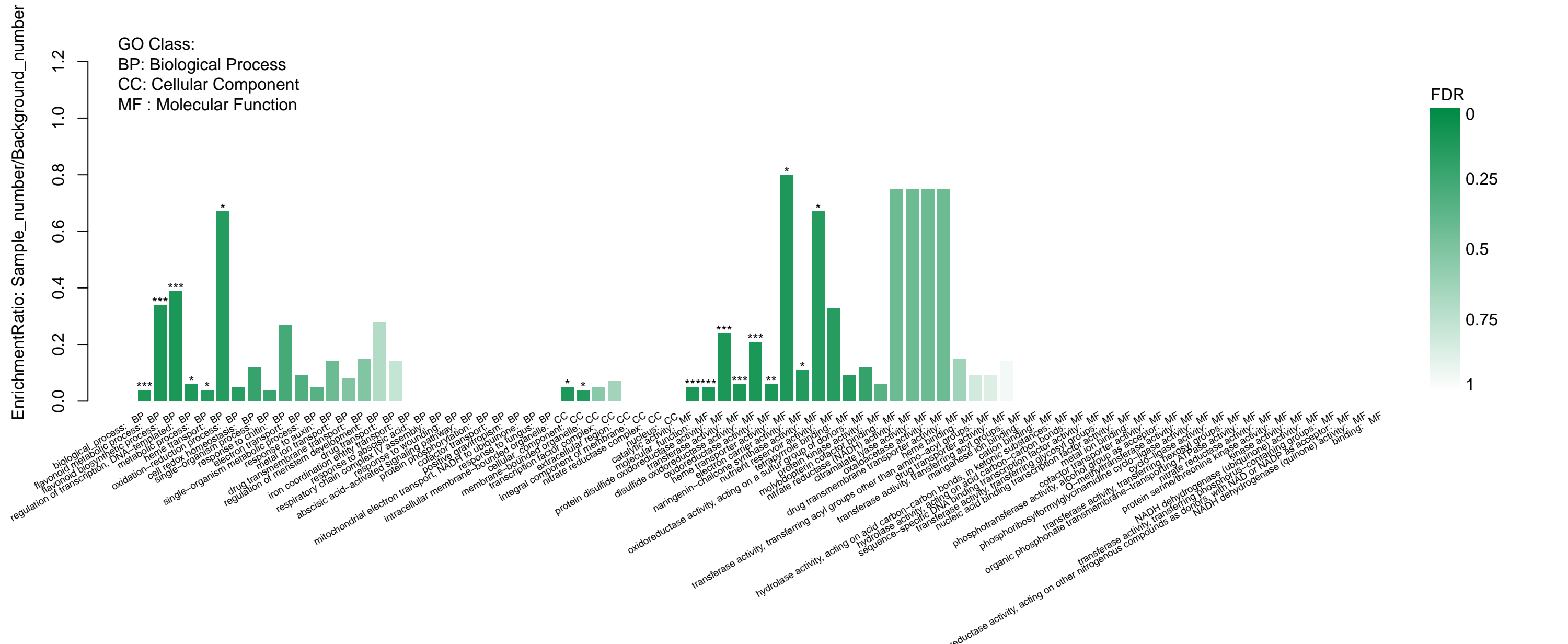

Supplement: S7 Fig — (PDF) [file pone.0164235.s007.pdf]

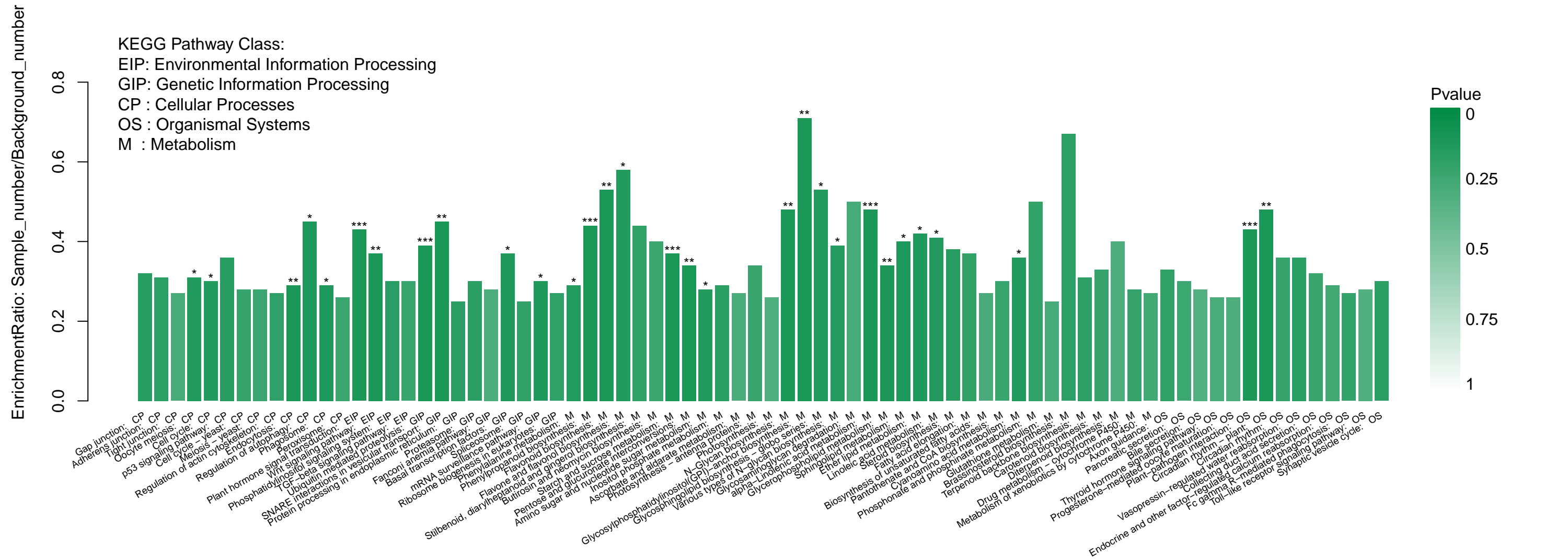

Supplement: S8 Fig — (PDF) [file pone.0164235.s008.pdf]

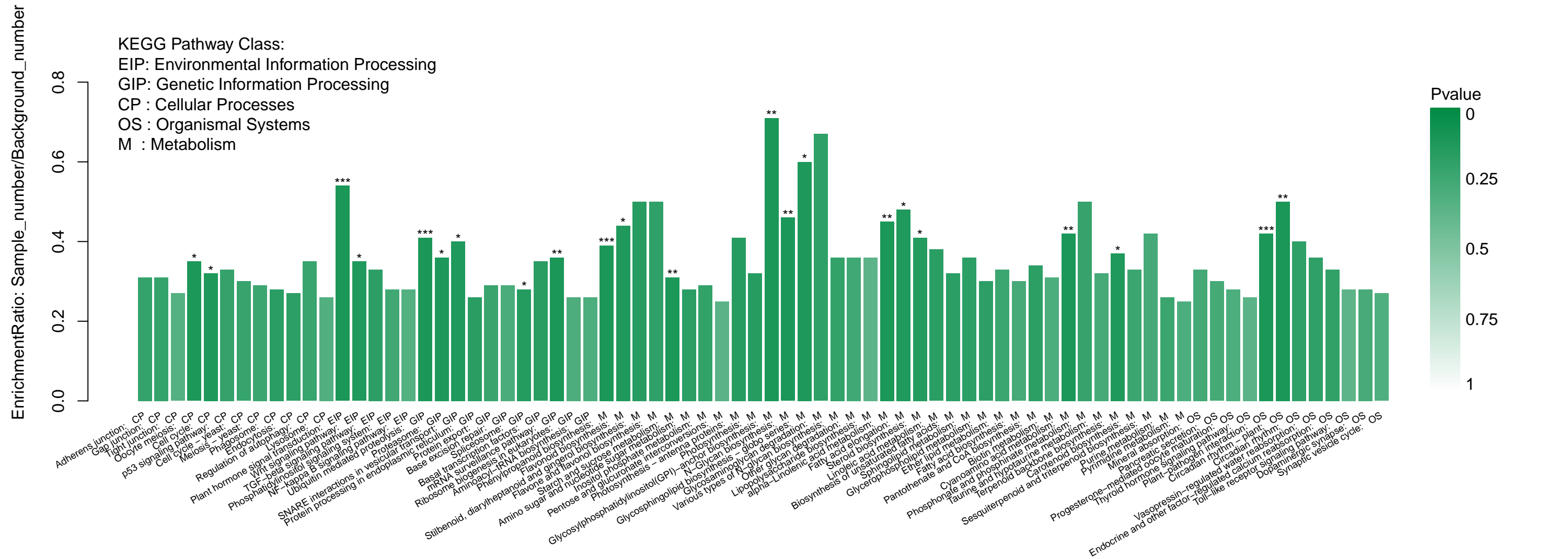

Supplement: S9 Fig — (PDF) [file pone.0164235.s009.pdf]

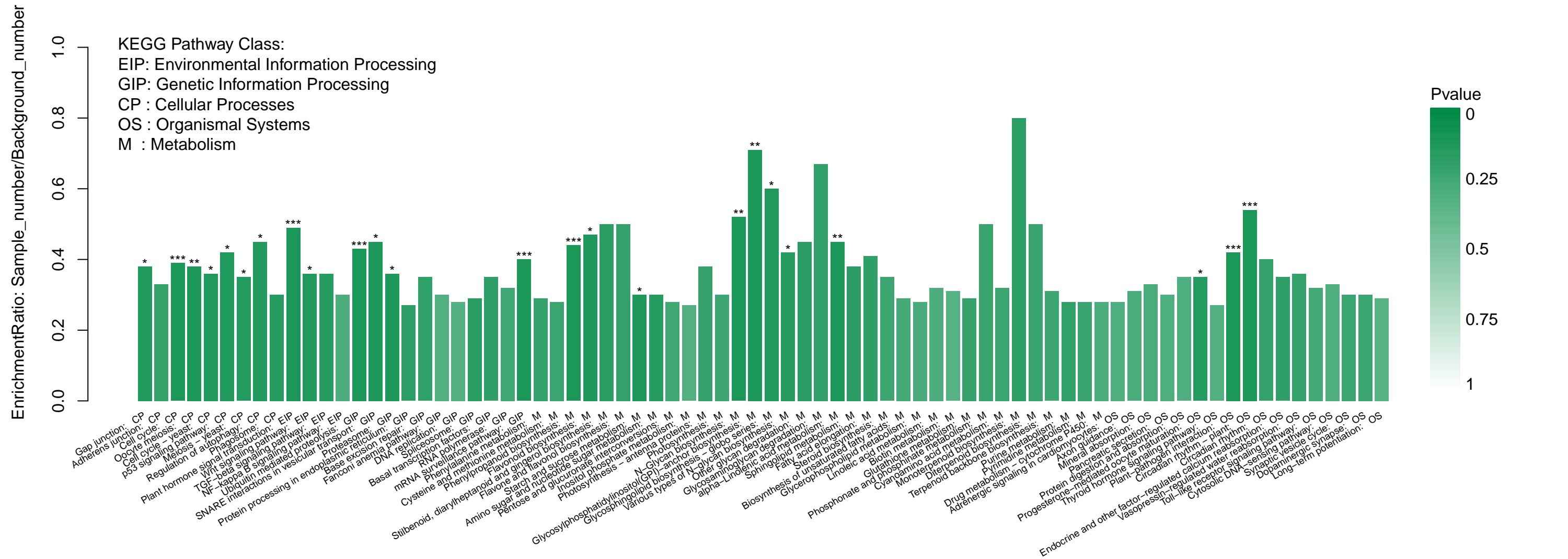

Supplement: S10 Fig — (PDF) [file pone.0164235.s010.pdf]
