# Supplementary material for: De Novo Sequencing and Assembly Analysis of the Pseudostellaria heterophylla Transcriptome
Source: PLoS One. 2016 Oct 20;11(10):e0164235. doi: 10.1371/journal.pone.0164235 (PMC5072632; doi:10.1371/journal.pone.0164235)
Supplement: S4 Fig — (PDF) [file pone.0164235.s004.pdf]

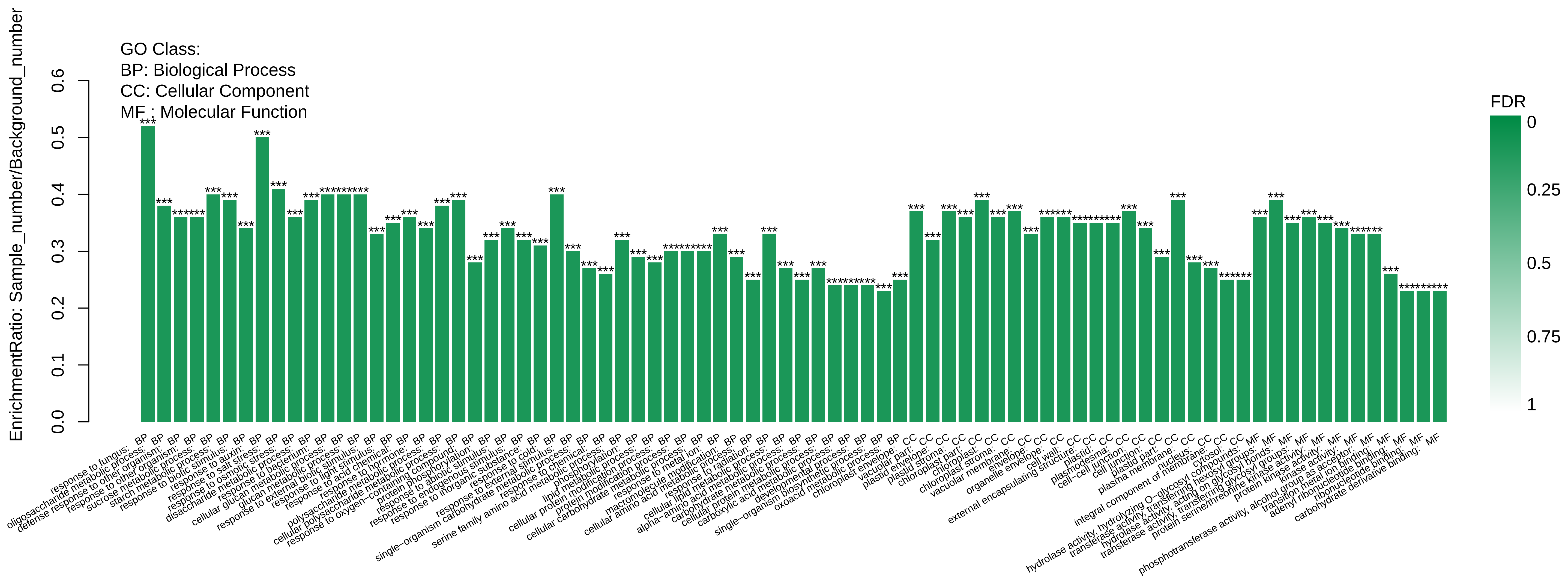
GO Class:
BP: Biological Process
CC: Cellular Component
MF : Molecular Function
EnrichmentRatio: Sample_number/Background_number
0.0
0.1
0.2
0.3
0.4
0.5
0.6
FDR
0
0.25
0.5
0.75
1
response to fungus: BP
oligosaccharide metabolic process: BP
defense response to other organism: BP
response to other organism: BP
sucrose metabolic process: BP
starch metabolic process: BP
response to biotic stimulus: BP
response to auxin: BP
response to salt stress: BP
response to osmotic stress: BP
disaccharide metabolic process: BP
response to bacterium: BP
cellular glucan metabolic process: BP
glucan metabolic process: BP
response to external biotic stimulus: BP
response to light stimulus: BP
response to acid chemical: BP
response to hormone: BP
polysaccharide metabolic process: BP
cellular polysaccharide metabolic process: BP
response to oxygen−containing compound: BP
protein phosphorylation: BP
response to abiotic stimulus: BP
response to endogenous stimulus: BP
response to inorganic substance: BP
response to cold: BP
response to external stimulus: BP
single−organism carbohydrate metabolic process: BP
response to chemical: BP
serine family amino acid metabolic process: BP
phosphorylation: BP
lipid metabolic process: BP
cellular protein modification process: BP
protein modification process: BP
cellular carbohydrate metabolic process: BP
response to metal ion: BP
macromolecule modification: BP
cellular amino acid metabolic process: BP
response to radiation: BP
cellular lipid metabolic process: BP
alpha−amino acid metabolic process: BP
carbohydrate metabolic process: BP
cellular protein metabolic process: BP
carboxylic acid metabolic process: BP
developmental process: BP
single−organism biosynthetic process: BP
oxoacid metabolic process: BP
chloroplast envelope: CC
vacuolar part: CC
plastid envelope: CC
plastid stroma: CC
chloroplast part: CC
chloroplast: CC
chloroplast stroma: CC
vacuolar membrane: CC
envelope: CC
organelle envelope: CC
external encapsulating structure: CC
cell wall: CC
plastid: CC
plasmodesma: CC
cell−cell junction: CC
cell junction: CC
plastid part: CC
plasma membrane: CC
nucleus: CC
integral component of membrane: CC
cytosol: CC
hydrolase activity, hydrolyzing O−glycosyl compounds: MF
transferase activity, transferring hexosyl groups: MF
hydrolase activity, acting on glycosyl bonds: MF
transferase activity, transferring glycosyl groups: MF
protein serine/threonine kinase activity: MF
protein kinase activity: MF
phosphotransferase activity, alcohol group as acceptor: MF
kinase activity: MF
transition metal ion binding: MF
adenyl ribonucleotide binding: MF
ribonucleotide binding: MF
carbohydrate derivative binding: MF
