# Supplementary material for: De Novo Sequencing and Assembly Analysis of the Pseudostellaria heterophylla Transcriptome
Source: PLoS One. 2016 Oct 20;11(10):e0164235. doi: 10.1371/journal.pone.0164235 (PMC5072632; doi:10.1371/journal.pone.0164235)
Supplement: S11 Fig — (PDF) [file pone.0164235.s011.pdf]

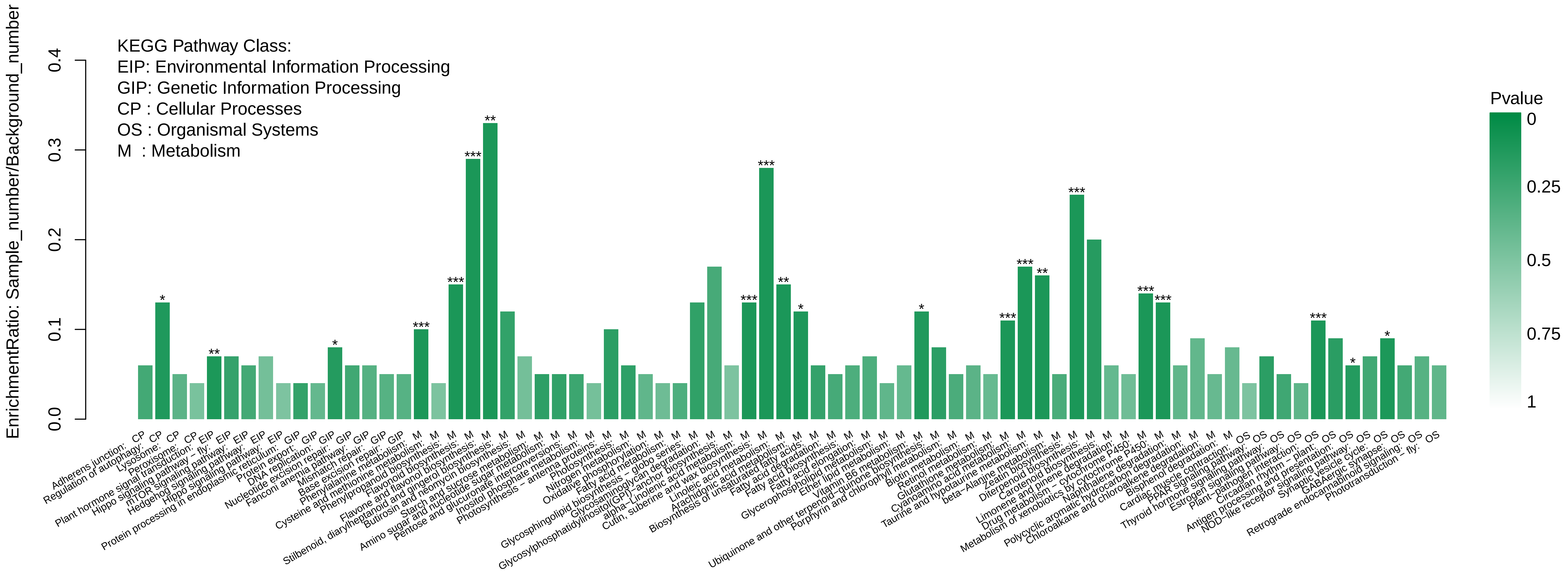
KEGG Pathway Class:
EIP: Environmental Information Processing
GIP: Genetic Information Processing
CP : Cellular Processes
OS : Organismal Systems
M : Metabolism
EnrichmentRatio: Sample_number/Background_number
0.0
0.1
0.2
0.3
0.4
Pvalue
0
0.25
0.5
0.75
1
Adherens junction: CP
Regulation of autophagy: CP
Lysosome: CP
Peroxisome: CP
Plant hormone signal transduction: EIP
Hippo signaling pathway − fly: EIP
mTOR signaling pathway: EIP
Hedgehog signaling pathway: EIP
Hippo signaling pathway: EIP
Protein processing in endoplasmic reticulum: GIP
Protein export: GIP
DNA replication: GIP
Nucleotide excision repair: GIP
Fanconi anemia pathway: GIP
Mismatch repair: GIP
Base excision repair: GIP
Cysteine and methionine metabolism: M
Phenylalanine metabolism: M
Phenylpropanoid biosynthesis: M
Flavonoid biosynthesis: M
Flavone and flavonol biosynthesis: M
Stilbenoid, diarylheptanoid and gingerol biosynthesis: M
Butirosin and neomycin biosynthesis: M
Starch and sucrose metabolism: M
Amino sugar and nucleotide sugar metabolism: M
Pentose and glucuronate interconversions: M
Inositol phosphate metabolism: M
Photosynthesis − antenna proteins: M
Photosynthesis: M
Nitrogen metabolism: M
Oxidative phosphorylation: M
Fatty acid metabolism: M
Glycosphingolipid biosynthesis − globo series: M
Glycosaminoglycan degradation: M
Glycosylphosphatidylinositol(GPI)−anchor biosynthesis: M
alpha−Linolenic acid metabolism: M
Cutin, suberine and wax biosynthesis: M
Linoleic acid metabolism: M
Arachidonic acid metabolism: M
Biosynthesis of unsaturated fatty acids: M
Fatty acid degradation: M
Fatty acid biosynthesis: M
Glycerophospholipid metabolism: M
Fatty acid elongation: M
Ether lipid metabolism: M
Vitamin B6 metabolism: M
Ubiquinone and other terpenoid−quinone biosynthesis: M
Porphyrin and chlorophyll metabolism: M
Biotin metabolism: M
Retinol metabolism: M
Glutathione metabolism: M
Cyanoamino acid metabolism: M
Taurine and hypotaurine metabolism: M
beta−Alanine metabolism: M
Zeatin biosynthesis: M
Diterpenoid biosynthesis: M
Carotenoid biosynthesis: M
Limonene and pinene degradation: M
Drug metabolism − cytochrome P450: M
Metabolism of xenobiotics by cytochrome P450: M
Naphthalene degradation: M
Polycyclic aromatic hydrocarbon degradation: M
Chloroalkane and chloroalkene degradation: M
Bisphenol degradation: M
Cardiac muscle contraction: OS
Thyroid hormone signaling pathway: OS
PPAR signaling pathway: OS
Estrogen signaling pathway: OS
Plant−pathogen interaction: OS
Circadian rhythm − plant: OS
Antigen processing and presentation: OS
NOD−like receptor signaling pathway: OS
Synaptic vesicle cycle: OS
Retrograde endocannabinoid signaling: OS
GABAergic synapse: OS
Phototransduction − fly: OS
